# Strategies for the Synthesis of Yardsticks and Abaci for Nanometre Distance Measurements by Pulsed EPR

**DOI:** 10.3390/molecules191220227

**Published:** 2014-12-03

**Authors:** Silvia Valera, Bela E. Bode

**Affiliations:** EaStCHEM School of Chemistry, Biomedical Sciences Research Complex and Centre of Magnetic Resonance, University of St Andrews, KY16 9ST Fife, UK

**Keywords:** model systems, synthesis, EPR distance measurements, nitroxide and trityl radicals, metal centres

## Abstract

Pulsed electron paramagnetic resonance (EPR) techniques have been found to be efficient tools for the elucidation of structure in complex biological systems as they give access to distances in the nanometre range. These measurements can provide additional structural information such as relative orientations, structural flexibility or aggregation states. A wide variety of model systems for calibration and optimisation of pulsed experiments has been synthesised. Their design is based on mimicking biological systems or materials in specific properties such as the distances themselves and the distance distributions. Here, we review selected approaches to the synthesis of chemical systems bearing two or more spin centres, such as nitroxide or trityl radicals, metal ions or combinations thereof and outline their application in pulsed EPR distance measurements.

## 1. Introduction

Electron Paramagnetic Resonance (EPR) or Electron Spin Resonance (ESR) is a spectroscopic technique sensitive to paramagnetic centres, such as metal ions with unpaired electrons or organic free radicals. Pulsed EPR techniques have greatly increased the amount of accessible information [1,2], and in particular distance measurements between two or more paramagnetic centres have attracted increasing attention as complementary tools for structural studies [3,4].

Pulsed EPR experiments give access to distances between endogenous paramagnetic centres, like paramagnetic metal centres [5] and cofactor radicals [6,7], or spin-labels chemically introduced by site-directed spin-labelling (SDSL) [3,8,9]. Most commonly nitroxides spin-labels are introduced viasulfur-specific chemistry linking to cysteine residues. The latter can either be native or generated by site-directed mutagenesis [3]. In most cases structural and functional perturbation introduced by nitroxide spin-labelling is minor [4].

EPR distance measurements are generally based on the quantification of the magnetic dipole-dipole coupling between the spins. This coupling frequency is directly related to the inter-spin distance. In the secular and point dipole approximations this frequency is given by Equation (1) [10,11]:

(1) $$\nu_{dd}=\;\frac{\mu_{0}\mu_{B}^{2}}{8\pi^{2}\hbar }\;\frac{g_{A}g_{B}}{r_{AB}^{3}}\;(1-3{\text{cos}}^{2}\theta_{AB})$$

where ν_*dd*_ is the dipole-dipole splitting in frequency units, μ_0_ is the vacuum permeability, μ_*B*_ is the Bohr magneton, *g_A_* and *g_B_* are the g-factors of the electron spins, ħ is Plank’s constant divided by 2π, *r_AB_* is the distance between two spins and θ*_AB_* is the angle between the distance vector connecting the two spins and the external magnetic field B_0_.

For the interaction between two organic radicals (*g_A_* = *g_B_* = 2.005) this is calculated by Equation (2) [12]:

(2) $$r_{AB}[nm]=\sqrt[{3}]{\frac{52.18[MHz]}{\nu_{dd}}}(1-3{\text{cos}}^{2}\theta_{AB})$$

However, in a powder or glassy frozen solution the dipolar splitting is dominated by the most probable θ*_AB_* of θ*_┴_* = 90°.

Information on the inter-spin distances can be extracted by continuous wave (CW) or pulsed EPR experiments such as Pulsed Electron-Electron Double Resonance (PELDOR - also known as Double Electron-Electron Resonance or DEER) [13] or Double Quantum Coherence (DQC)-filtered EPR [14,15,16]. In CW EPR this is often estimated via spectral broadening which is sensitive for distances up to 2 nm [17,18]. This upper distance limit is dictated by the spectral line width. The corresponding dipolar splitting is approximately 6 MHz and commonly not resolved in anisotropic nitroxide EPR spectra, thus, making precise quantification of inter-spin distances challenging. On the other hand, the pulsed techniques are based on the coherent detection of the dipolar interaction [4]. Here, the upper distance limit is commonly specified to 8 nm. For measuring this distance at least 10 µs observation time (one full period of coherent evolution) would be required. This is challenging to achieve even in favourable cases due to relaxation phenomena. For technical aspects of the EPR methods see, for example, Schiemann *et al*. [3] and Jeschke *et al*. [19]. 

PELDOR in combination with SDSL has attracted increasing attention from the field of structural biology [4,9]. Recently, DQC in combination with stable trityl radicals has been demonstrated as a feasible alternative method that allows measurements on biological systems, even at ambient temperatures [20]. Even though PELDOR requires a second microwave frequency it is more frequently applied than DQC. This is commonly attributed to the higher robustness towards experimental imperfections.

Pulsed EPR distance measurements do not only give information on inter-spin distances, but also provide powerful geometric constraints for structure determination of biomolecules such as angular correlations [21,22], structural flexibility [23,24,25] and the number of interacting spins [26,27,28,29], in the presence or absence of electron-electron exchange couplings (*J*) [30,31]. While these additional effects potentially provide information beyond distances on the system under study, they can complicate structure elucidation in complex (bio)molecules. The extent of these contributions is often unknown beforehand. Furthermore, ignoring additional contributions to the signal might lead to data misinterpretation. Since the development of pulsed experiments for EPR distance measurements, synthetic efforts have been made to design systems for quantitatively studying orientation selection, multi-spin effects or exchange couplings. Model compounds have played a fundamental role in testing the design of EPR experiments and validating their precision and accuracy [4,32].

Herein, we review a selection of strategies for the synthesis of chemical systems bearing two or more nitroxide radicals or alternative spin centres at nanometre distances together with key applications. Similar molecules have become of major importance in hyperpolarisation techniques such as microwave-assisted dynamic nuclear polarisation.

## 2. Spin-Labels

**Figure 1:**
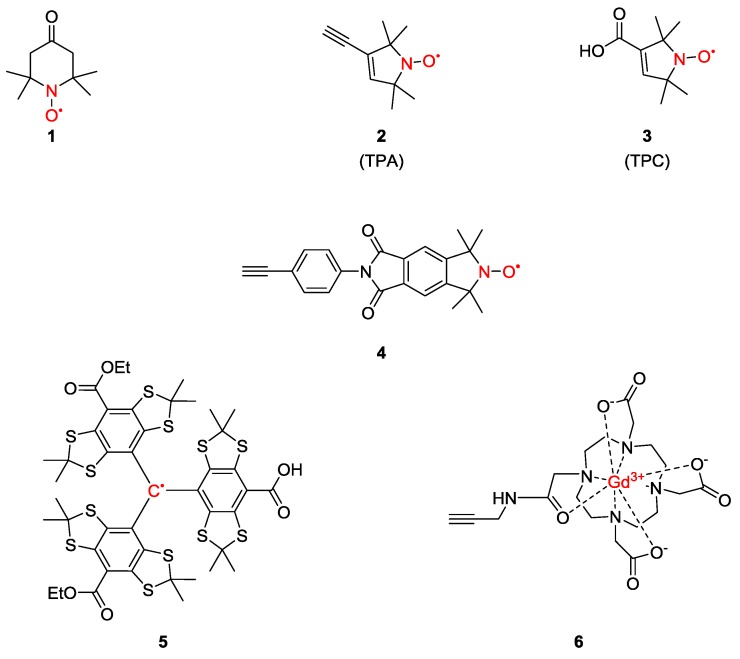
Spin-labels commonly used for model systems.

Spin-labels are paramagnetic centres with a functionality which allows their attachment to the system under study. Paramagnetic centres used for spin-labelling include stable organic radicals such as nitroxides **1**–**4**, trityl radicals **5** or metal centres **6** in the form of ions or clusters (Figure 1) [33,34]. The latter can be attached to the system or replace native, diamagnetic metal ions [35]. Design of spin-labels relies on their stability, paramagnetic properties and reactivity which should not affect the systems’ structures or functions. However, the main challenge is ensuring the ease of introduction in biomacromolecules, such as nucleic acids and proteins [36]. 

The most commonly used stable radicals for EPR-based distance measurements are nitroxide radicals based on piperidine, pyrrolidine, pyrroline or imidazoline heterocycles [32]. This can be attributed to their relative stability to redox reactions and ease of isolation and handling. To-date the most commonly used stable radicals for pulsed EPR model compounds are the pyrroline-based spin-labels **2** and **3**. These two spin-labels differ in the functionality used for linking them. The carboxylic acid functional group of **3** allows its attachment to a free hydroxyl group through esterification under mild conditions [37,38]. On the other hand, **2** is commonly cross-coupled to an aryl or vinyl halide using Sonogashira conditions. In principle, the two methods for attachment are orthogonal. However, increasing interest for *in vivo* pulsed EPR has recently fuelled the need for more stable spin-labels as tetramethylpyrroline-based nitroxides that exhibit half-lives of minutes under these conditions [39]. Recently, trityl radicals [40] and gadolinium(III)-based complexes [41] have been introduced as promising spin labels for in-cell measurements and both have already been used as labels for complex biological structures [20,41]. Furthermore, trityl-based spin-labels allow DQC distance measurements with standard hardware [40], while Gd-based systems offer increased sensitivity using high frequency EPR [35].

When choosing the strategy for spin-labelling both the structure of the linking group and the chemistry for its attachment have to be considered. The intrinsic flexibility of the spin-label as well as the conformational flexibility of the linking group can add to the width of the EPR distance distribution between the attachment sites. In particular cases, the choice of labelling strategy can be limiting. Furthermore, the labelling chemistry should be highly selective under mild conditions [37,38]. Thus, spin-labels with a variety of functional groups, for example alkyne (compounds **2**, **4** and **6**) and carboxylic acid (compounds **3** and **5**) are available for the syntheses of the desired model compounds.

## 3. Model Systems

Model systems have accompanied the development of pulsed EPR distance measurements [10,32]. These have been extensively used for calibration of pulsed EPR distance measurements, including dead-time free PELDOR/DEER [42,43], a single-frequency technique for refocusing (SIFTER) [44] and DQC [14,45]. In addition, the potential for distance measurement applications involving native paramagnetic metal centres or their introduction by ion exchange or SDSL has driven design of the corresponding simplified chemical models. Owing to their use as benchmarks for the precision and accuracy of distance measurements and spin counting, biradical and polyradical model systems are sometimes referred to as ‘yardsticks’ and ‘abaci’. The use of model systems with a known chemical structure gives an easy access to distance predictions via increment models [46] or molecular dynamics simulations [25].

Biradical and polyradical model systems are commonly approached by spin-labelling of a spacer or scaffold molecule. Here, two or more spin-labels are linked to the appropriate functionalities present on the spacer. Typical examples are reactions between scaffolds bearing hydroxylated or aryl halide functionalities and carboxylic acids or terminal alkynes present on the spin-labels, respectively.

Desirable properties of yardsticks include high solubility in glass-forming solvents and a well-defined structural rigidity. For this reason, poly(*para*-phenyleneethynylene)s have been extensively used as scaffolds for two-spin systems on length-scales from 1.4 [31] to 8 nm [25]. This class of building blocks displays a limited structural and conformational flexibility, thus, allowing measurement of well-defined inter-spin distances [23,25,38]. Both linear and bent rigid systems, consisting of phenylacetylene or biphenyl units [24,26,27,37,38], aliphatic chains [29,47] and condensed rings [30] have been reported.

Model systems bearing more than two spins, which typically consist of a phenyleneethynylene- or biphenyl-based backbone with up to four nitroxide moieties [27,37] attached, have been designed for quantifying multi-spin effects [26]. Furthermore, systems bearing two metal ions as paramagnetic centres [48,49] or their combination with nitroxide radicals [50,51] have been used for the optimisation of distance measurements involving these spectroscopically challenging spin centres. Biologically relevant examples include hemes [52] and iron-sulfur clusters [5]. Furthermore, biomaterial-inspired yardsticks such as bis-peptide molecular rods [53] or double-helical DNAs [54,55] have been reported. However, in the following the focus will be on syntheses and structures of purely synthetic model systems.

### 3.1. Biradicals

As the majority of experiments aim to extract a single distance between two electron spins the prototypical model compound is a biradical with a very narrow distribution of distances between the paramagnetic centres. These represent the most abundantly used model systems for pulsed EPR distances [23,25]. Additionally, biradicals introducing aspects such as increased backbone flexibility [29,47], ferromagnetic and antiferromagnetic electron-electron exchange couplings [30,31] and distance measurements in the strong coupling regime [56] have all been studied by pulsed EPR distance measurements.

#### 3.1.1. Nitroxide-Based Biradicals

**Figure 2:**
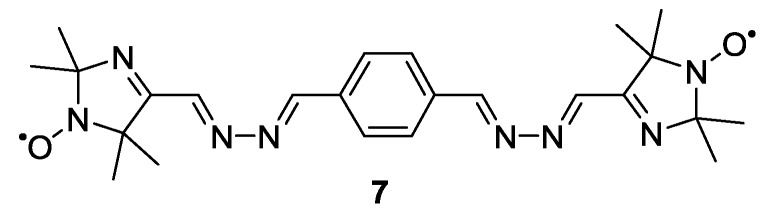
Imidazole-based biradical.

Milov *et al.* reported one of the first examples of biradical model systems on which the basic principles of PELDOR distance measurements were studied [10]. Imidazole-based biradical **7** [57] (Figure 2) has been used for testing further new experiments such as field-step ELDOR. Here, the requirement of a second frequency source is avoided by switching the magnetic field. Due to challenging hardware requirements the method is not used widely [58]. Furthermore, **7** was employed to test the relaxation-induced dipolar modulation enhancement (RIDME) experiment [59]. This distance measurement is based on relaxation processes rather than microwave pulses for the inversion of coupled spins. The method has been applied at high field [60] and improved to a dead-time free variant [61].

Imidazole-based nitroxide radicals have been the spin-label of choice for the very first model systems for EPR distance measurements [10] before **2** and **3** were established. The latter have the unpaired electron localised on the N-O bond which is advantageous especially for the accuracy of measurements of short radical-radical distances [38]. For this reason later model system examples bear pyrroline or piperidine-based nitroxides [32].

**Scheme 1:**
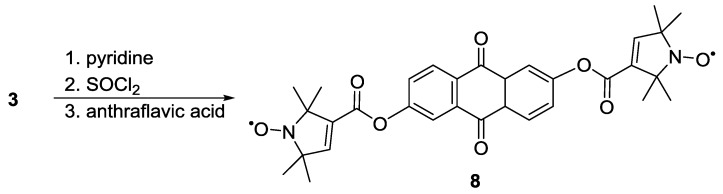
Esterification of anthraflavic acid to spin-label **3**.

Another early example of a model system synthesised for calibration of pulsed EPR distance measurements is the anthraflavic acid-based biradical **8**, reported by Larsen and Singel [21]. The synthesis of the rigid biradical (Scheme 1) used conditions similar to those previously reported by Rosantsev [62].

The PELDOR traces obtained from **8** are among the first to show an electron-electron dipolar modulation. This seminal study not only demonstrated the oscillation frequency to give direct access to the size of the dipolar coupling, thus inter-spin distance, but also showed the first consideration of orientation selection in PELDOR. The distance and geometry deduced from pulsed EPR were found to be in excellent agreement with a planar structural model. The opportunity to study mutual orientations by looking at a number of independent spectral positions proved the method to be of valuable potential for spin-labelled biological systems [21].

**Figure 3:**
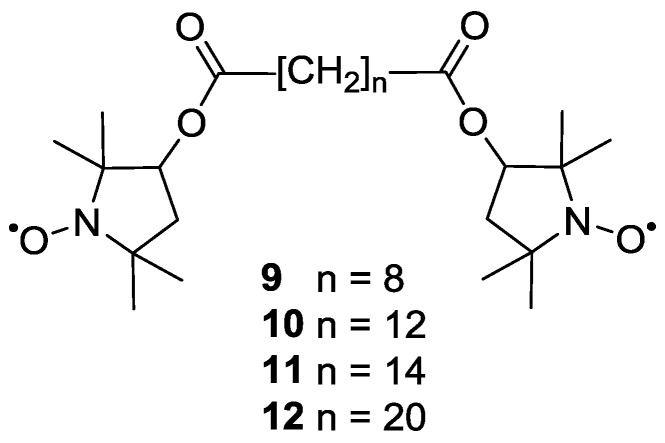
Structure of flexible biradical systems.

Pfannerbecker *et al.* [47] reported distance measurements on both rigid and flexible biradicals. The rigid system **8** was used as a reference for end-to-end distances and for determination of optimal experimental parameters. The flexible systems **9**–**12** with increasing numbers of methylene units (Figure 3) were used for studying conformational statistics and the resulting distributions of end-to-end distances. The experiments proved the reliability of pulsed EPR for the extraction of distances in systems with large backbone flexibility.

**Scheme 2:**
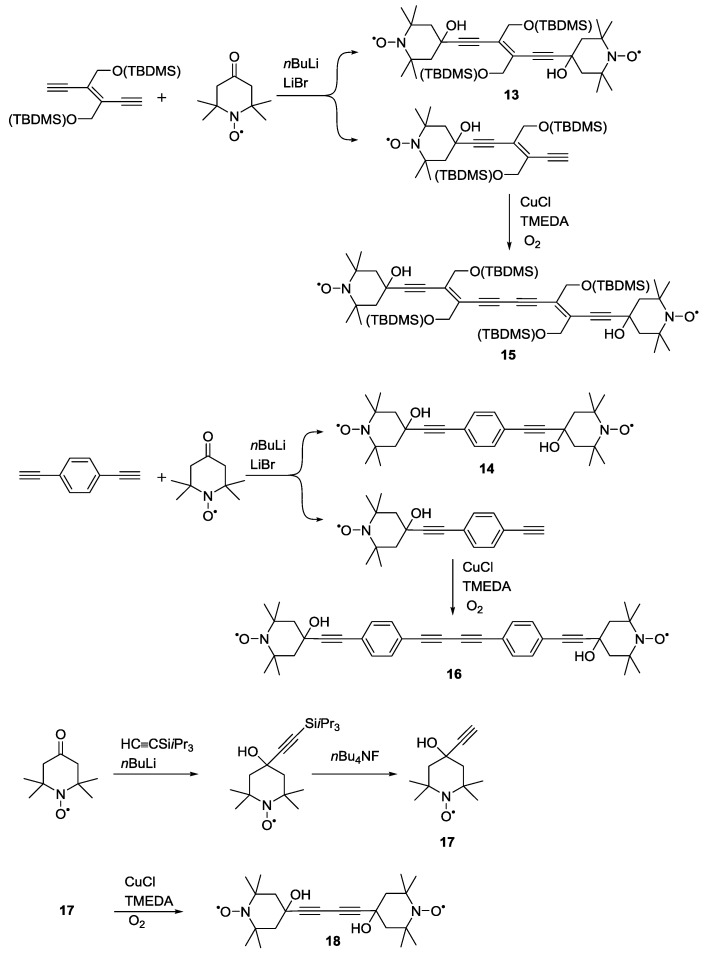
Synthesis of a series of biradical model systems based on oxidative Hay couplings.

Scheme 2 shows representatives of an early, modular approach to the synthesis of biradicals with different spin-spin distances and narrow distance distributions, for pulsed EPR distance measurements [43]. It was speculated that the application of these methods for quantitative determination of unknown structures was limited by technical shortcomings - mainly experimental dead-time - and by the lack of a demonstration via a series of measurements of well-defined end-to-end distances well into the nanometre range.

TMEDA: *N*,*N*,*N*',*N*'-tetramethylethylenediamine, TBDMS= *tert*-butyldimethylsilyl.

A convergent synthesis gave access to a series of biradicals with modelled distances of 1.4, 1.7, 1.9, 2.4 and 2.8 nm. The labelling was based on the reaction of 4-TEMPONE with acetylides. The *n*-BuLi and LiBr-mediated reaction of 4-TEMPONE with spacer building blocks bearing two terminal alkynes gave access to both biradicals **13** and **14**. This reaction also yielded the corresponding singly-labelled building blocks that were then dimerised via oxidative Hay coupling to give biradicals **15** and **16**. Compound **17** could be obtained by reaction of 4-TEMPONE with triisopropylsilylethyne in presence of *n*-BuLi and subsequent deprotection. Biradical **18** was obtained from the dimerization of **17**. Distance measurements performed on these systems have marked two important advances in pulsed EPR: they have shown the first application of a series of biradicals with well-defined end-to-end distances to demonstrate the accuracy of pulsed distance measurements. The measured distances were found to be in agreement, within 0.2 nm error, with the ones modelled by molecular dynamics. Secondly, Martin *et al*. extended the pulse sequence for this experiment from the original three-pulse sequence to a four-pulse sequence, thus, introducing the dead-time free experiment which is the most commonly used experiment for quantitative distance measurements to date [43]. This newly developed technique has allowed reliable measurement of broad electron-electron coupling distributions [63].

**Scheme 3:**
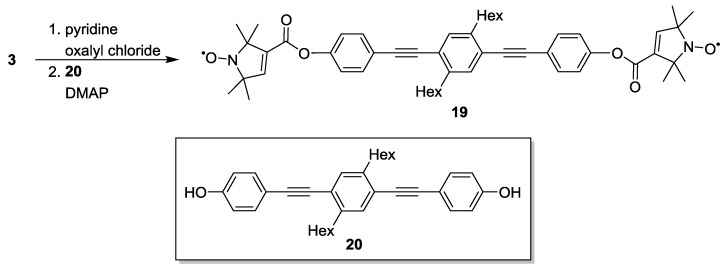
Synthetic path for synthesis of **19** using oxalyl chloride and pyridine.

Pannier *et al*. [42] used **8**, **16** and **19** (Scheme 3) to calibrate the dead-time free experiment. The newly reported rigid biradical **19** was synthesised from a terminally hydroxylated poly(phenyleneethynylene) backbone **20** which was esterified to two equivalents of **3**. The reaction using oxalyl chloride and pyridine, proceeds via the intermediate formation of the acyl chloride, thus facilitating the formation of the diester. The authors chose pyrroline-based **3** as spin-bearing group rather than the previously reported six-membered piperidine-based nitroxides. **3** exhibits a reduced flexibility and its carboxylic acid function allows formation of ester linkages.

Hex: *n-*hexyl (C_6_H_13_).

**Scheme 4:**
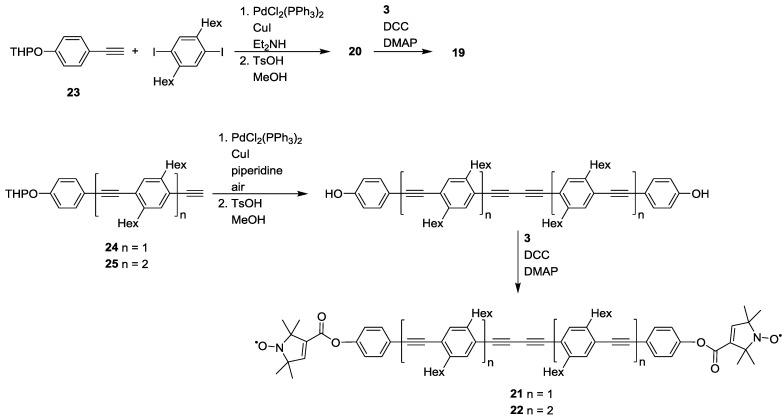
Synthesis of biradical model systems based on poly(phenyleneethynylene)-backbones.

DCC: dicyclohexylcarbodiimide; DMAP: *N*,*N*-dimethylaminopyridine.

To streamline the synthesis of a series of biradical yardsticks Godt *et al.* reported a convenient route for accessing linear biradicals with inter-spin distances from 2.8 to 5.2 nm as well as for generating a star-shaped three spin abacus [38]. The rod-like biradicals **19**, **21** and **22** bear poly(phenyleneethynylene) backbones. The spacers were synthesized by Sonogashira cross-couplings of substituted iodo- or diiodo-benzenes with a terminal alkyne building block bearing a tetrahydropyranyl (THP) protected phenol, like **23**. Building-blocks of different lengths such as **24** and **25** were generated via addition of further segments by Sonogashira couplings. The free phenols could be isolated after removal of the THP protective group. Compound **3** was attached to the phenols via Steglich esterification [64] (Scheme 4). The same route has allowed the introduction of deuteration and ^15^N labelling via ^2^H and/or ^15^N-labelled **3** [65].

**Figure 4:**
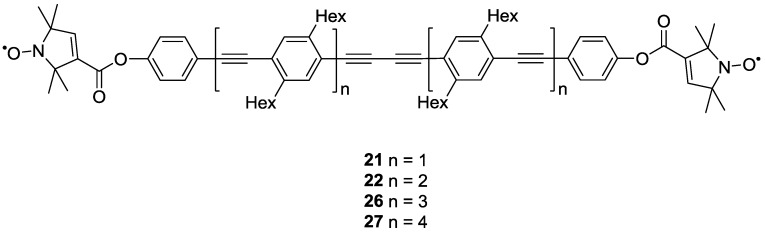
Poly(phenyleneethynylene)-based biradical yardsticks.

The same concept of using poly(*para*-phenyleneethynylene)-units as building-blocks for the synthesis of molecular rods with increasing lengths has later been expanded by the same groups [25]. The synthesis that yielded **21** and **22** [38] was expanded to compounds **26** and **27** (Figure 4). This series of yardsticks has inter-spin distances of up to 7.5 nm.

Distance measurements on **19**, **21**, **22**, **26** and **27** allowed quantification of the intrinsic flexibility of functional nanostructures bearing poly(*para*-phenyleneethynylene)-backbones. This involved the separation of the contribution of the spin-labels themselves to the distance distributions. This separation was achieved by distance measurements on the series of model systems and allowed extracting the flexibility of the spacer, which is representative of *para*-phenyleneethynylene polymers [25].

The same compounds have been used to validate a series of approaches for data processing [63,66,67] and to test new experimental schemes as for example a variable time variant of PELDOR/DEER [68]. The sensitivity enhancement allowed precise measurement of a 7.5 nm distance in **27** [68]. However, this method is less robust and not recommended for general use [19]. Additionally **19**, **21** and **22** have been used for the validation of single-frequency methods for the detection of dipolar couplings [44].

**Figure 5:**
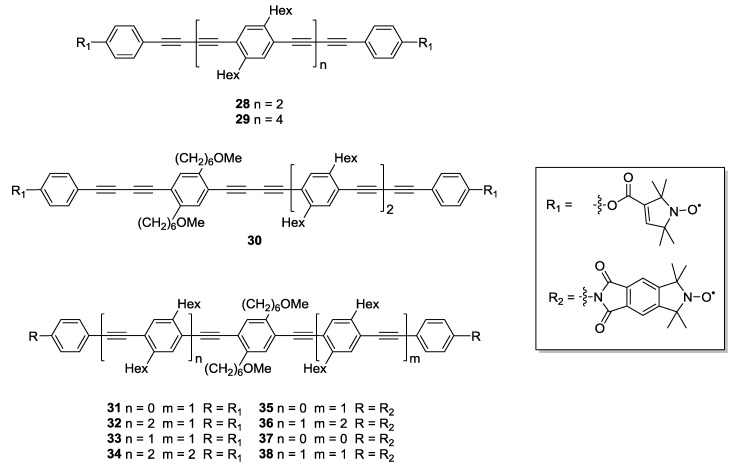
Oligo(*para*-phenyleneethynylene)- and oligo(*para*phenylenebutadiynylene)-based biradical yardsticks.

Further studies on the rigidity of polymer building-blocks have been performed by the same groups [23]. After having isolated the contribution of the spin label to the overall flexibility observed [25], the authors addressed a number of residual points. On the one hand, the temperature dependence of the persistence length was studied. Furthermore, the central butadiynylene in **21**, **22**, **26** and **27** might differ in flexibility from ethynylene units. To this end, a series of oligo(*para*-phenylenebutadiynylene)- (**28**–**30**) and oligo(*para*-phenyleneethynylene)-based (**31**–**38**) biradicals with varying inter-spin distances were synthesised using similar synthetic strategies to those previously reported [25,38,69]. Variation in the model systems’ structures has also been introduced by alternating **3** with conformationally unambiguous spin-label **4** [23,69], as reported in Figure 5. Compound **4** was chosen as alternative to the more commonly used **3** because of its axiality. All rotations around a linear linker will leave the N-O bond on the linker axis, thus, the impact of the spin label on the distance distribution is diminished.

The choice of alkyl or ether linkages in the side-groups played an important role for facilitating isolation of the desired building-blocks. Introduction of a more polar side chain in one building-block allows chromatographic separation of different homo- and cross-coupled products through their different polarities. This is especially significant as the Glaser coupling product is often found in Sonogashira cross-coupling reactions [23].

By combining simulation and experiment the determination of the conformational flexibility could be refined to higher accuracy. Additionally, the variation of the spin label allowed for an estimate of the error in separation of the contributions of spacer and label. Furthermore, the temperature dependence was investigated through the glass transition temperatures of the measurement matrices. As expected, higher glass transition temperatures resulted in reductions in the mean distances and broader distance distributions as evidenced by faster damping of dipolar oscillations [23].

**Figure 6:**
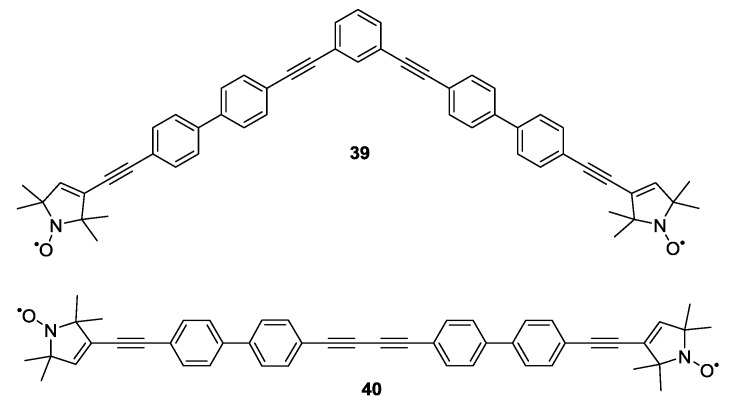
One linear and bent biradical.

Another example of biradical model systems based on phenylenes and ethynylenes has been reported by Margraf *et al.* [24]. Flexibilities were extracted from a bent (compound **39**) and a linear (compound **40**) biradical system as shown in Figure 6 by studying the orientational correlations between the nitroxides and distance vector.

The analysis of these orientation-selective X-band PELDOR traces has shown that in the presence of orientational correlations the conformational distribution of the molecule can be estimated [24]. The very same concept has later been employed determine the dominant dynamic mode for double-helical DNA [70]. In particular **40** has been used for calibration of a wide variety of experiments and data analysis approaches including the extraction of orientations [22], library approaches [71], out-of-phase PELDOR [72] or broadband inversion in PELDOR [73].

**Scheme 5:**
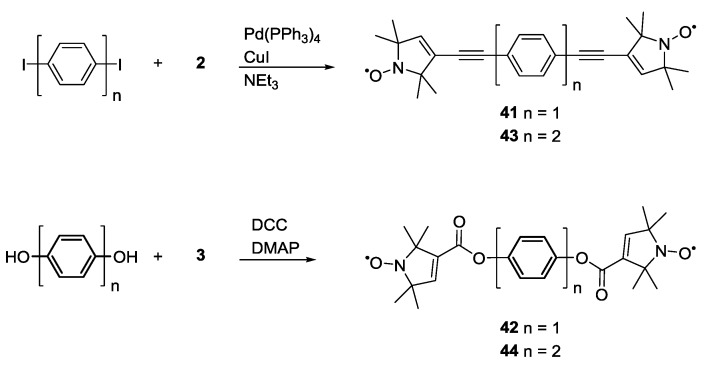
Synthesis of biradical model systems with variable spacer length and conjugation.

Furthermore, electron-electron exchange couplings have been quantified. This through-bond coupling mechanism can modify the frequency of the spin-spin coupling and, thus, affect distance measurements. To isolate and study this effect Weber *et al*. [31] reported the synthesis (Scheme 5) of biradicals **41**–**44**. Compounds **41** and **42** have similar inter-spin distances dictated by the presence of one single phenyl ring spacer. 

Similarly **43** and **44** have two phenyl rings as spacers. The only difference within these pairs of systems is the spin-label attached to the backbone: **2** in **41** and **43** or **3** in **42** and **44**. The first two were synthesized by Sonogashira cross-coupling reactions while **42** and **44** were obtained by Steglich esterifications

The presence of ester bonds in **42** and **44** was expected to diminish any through bond coupling, thus, they could be used as a reference for “zero exchange coupling systems”. The linkages via ethynylene-groups in **41** and **43** give rise to a through bond electron-electron exchange coupling between the radicals [57]. Distance measurements performed at S- and X-band frequencies (3 and 9 GHz, respectively) allowed discarding any hyperfine coupling contribution to the signal. The measurements have been exploiting orientation selection based on the anisotropic nitrogen hyperfine interaction and allowed separating the through space dipole-dipole coupling from the through bond exchange coupling [31]. 

**Scheme 6:**
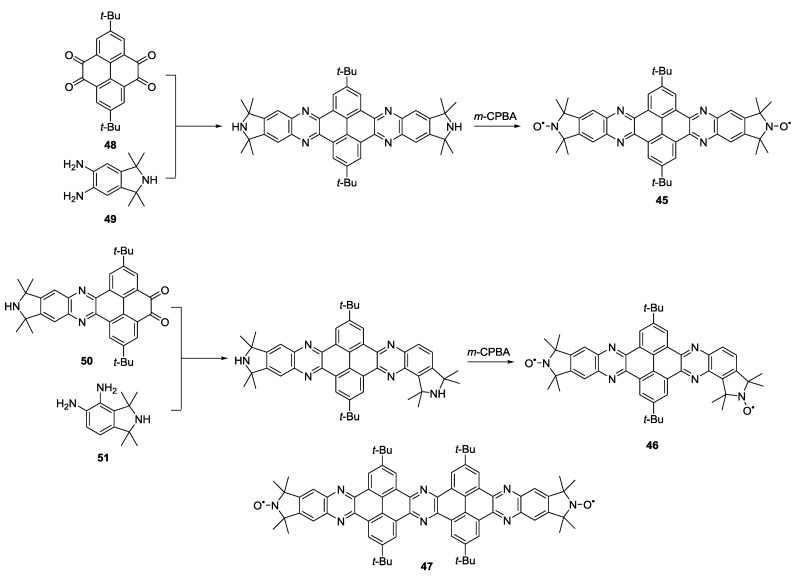
Synthesis of rigid biradicals with different substitution patterns.

Effects on distance measurements induced by the presence of electron-electron exchange coupling have also been studied on bis-nitroxides composed of annulated rings. Often rotational freedom blurs orientational correlations. The condensed structures of the model systems **45**, **46** and **47** reported by Margraf *et al.* [30] diminish rotational freedom of the nitroxides inducing very strong orientational correlations. Additionally, as for **41** and **43**, the full conjugation between the five-membered rings of the nitroxides gives rise to a non-vanishing electron-electron exchange coupling. Here, fixing the relative orientations of the nitroxides allowed using these molecules as benchmarks for the extraction of both ferromagnetic and antiferromagnetic exchange couplings. The pyrroline precursors of the nitroxides were attached to the conjugated system in two different substitution patterns giving rise to ferro- and antiferromagnetic interactions. The synthetic strategy, reported in Scheme 6, was based on condensation between **48** and **49**, followed by generation of the nitroxide radical using *m*-CPBA as the final step to give the target molecule **45**. A similar approach was used for the synthesis of **47** for which **50** and **51** were used as alternative starting materials yielding a different geometry and, thus, a different relative orientation of nitroxides. The earlier reported **48** [74] was used as a reference for orientation selection in rigidly labelled DNAs. The combination of simulations and the experimental EPR distance data allowed the separation of the exchange from the dipolar coupling. The rigidity of the molecules gave access to angular correlations as exploited in a later pulsed W-band (95 GHz) study [75].

**Figure 7:**
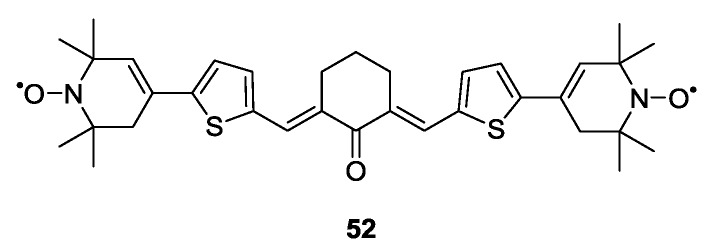
Structure of biradical used for calibration of DQC experiments.

Biradical model systems were used not only to optimise PELDOR distance measurements but also DQC experiments. **52** (Figure 7), for example, was used to design of an experiment for suppression of electron-nuclear effects when performing DQC in protonated matrices [76].

#### 3.1.2. Trityl-Based Biradicals

The combination of PELDOR distance measurements on model systems and quantitative simulations of the resulting data has proven the method to be precise and reliable for application to structurally unknown systems. DQC EPR [16] represents an alternative method for nanometre distance measurements. In comparison to PELDOR, DQC experiments are more sensitive. However, DQC requires full excitation of the EPR spectrum which means much more demanding hardware requirements in the case of nitroxides. Trityl radicals, on the other hand, have a comparatively narrow EPR line as they have vanishing *g*-anisotropy and only small hyperfine couplings to abundant isotopes. They have recently been considered as alternative spin-labels allowing DQC measurements in biological systems at room temperature [20]. Additionally, superior stability under reducing conditions compared to nitroxide radicals makes trityls interesting for in-cell applications [40].

**Scheme 7:**
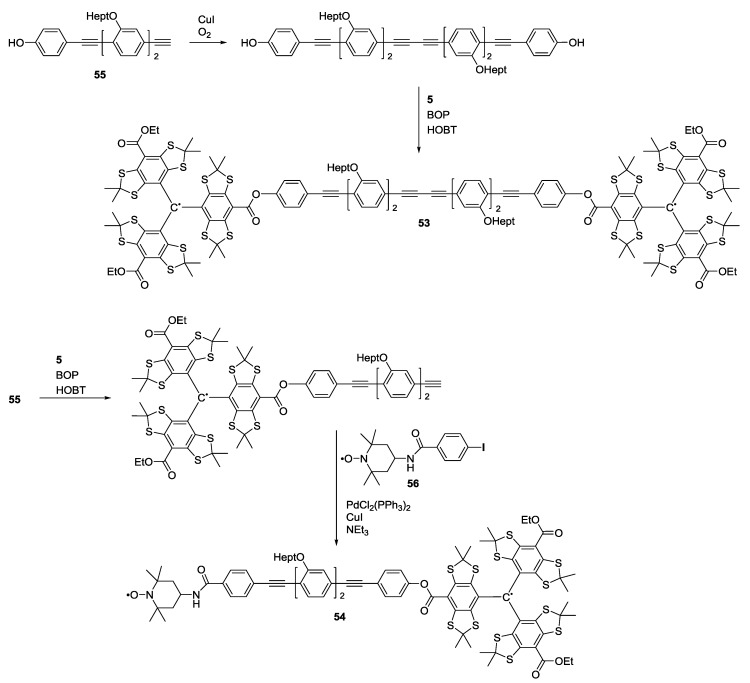
Trityl-trityl and trityl-nitroxide model systems.

Reginsson *etal.* reported the synthesis of two biradical model systems [40]. Compound **53** is a trityl biradical and **54** exhibits one trityl and one nitroxide radical (Scheme 7). Both systems are linked with **55** as spacer building-block. Compound **53** was obtained from esterifying the dimer of **55** to two equivalents of trityl radical **5** using benzotriazol-1-yloxytris(dimethylamino)phosphonium hexa-fluorophosphate (BOP) and 1-hydroxybenzotriazole (HOBT) as activating agents. Nitroxide radical **56** was coupled to the product of the esterification of **55** to **5** under standard Sonogashira conditions to give **54**. The rod-like spacers are composed of a series of acetylene and phenylene units with ether-linked alkyl side-chains to improve solubility. The model systems were designed to have well-defined inter-spin distances ranging from 3.5 (compound **53**) to 5 nm (compound **54**) well within the range of distances measured in biological systems.

**Scheme 8:**
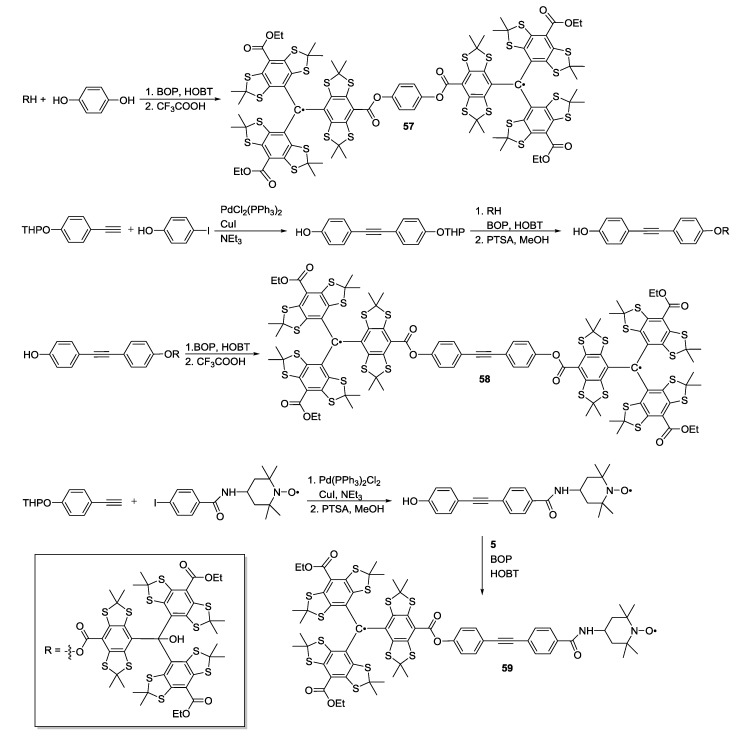
Synthesis of shorter distance trityl-trityl and trityl-nitroxide model systems.

The same authors extended the series by two further bis-trityl systems, **57** and **58**, and the trityl-nitroxide biradical **59** (Scheme 8) [56]. Cross-couplings were performed in presence of both free and THP-protected phenols. As opposed to the first series [40], trityl radicals in **57** and **58** were generated by reaction with trifluoroacetic acid after esterification.

Accurate trityl-trityl distances could be extracted by DQC measurements. However, for short distances the pseudo-secular part of the dipolar coupling and the spin density distribution within the trityl structure had to be taken into account. The resulting model systems’ inter-spin distances were measured to be 1.5 nm in **57**, 2.2 nm in **58** and 2.4 nm in **59**. These studies demonstrated the potential of DQC distance measurements for systems of unknown structure using commercial pulsed X-band EPR hardware [40,56].

### 3.2. Polyradicals

PELDOR distance measurements on systems containing more than two paramagnetic centres have been found to be challenging as the presence of multi-spin effects can hamper data interpretation [77,78]. Polyradical model systems have been developed mainly for two purposes: firstly, as abaci for proving the concept of ‘PELDOR spin counting’ [27,28,29] and secondly, for quantifying and potentially suppressing multi-spin contributions to the dipolar coupling [26,77,78]. Spin counting allows determining the number of interacting monomers in a multimer. On the other hand, multi-spin effects can complicate distance data analysis in systems with more than two unpaired electron spins.

For multi-spin model systems, the design of rigid spacers is often similar to those of yard-sticks. For example, backbones from phenylene- and ethynylene-units have been synthesised and spin-labels such as **2** [27], **3** [37,38] or **4** [69] have been attached. In order to uniquely identify multi-spin effects biradicals bearing the same general architecture but only two spin labels are often employed as reference molecules [26,27]. Thus, all effects only present in the multi-spin systems and not in the constituent biradicals can be isolated. 

**Scheme 9:**
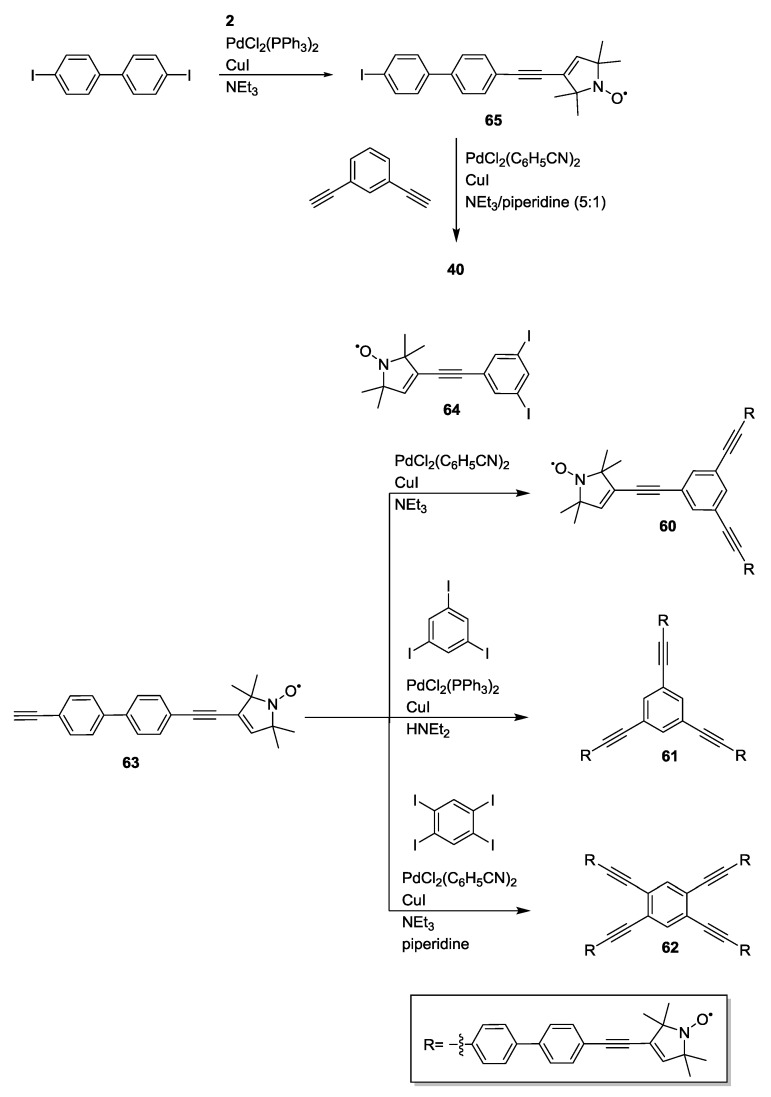
Convergent synthesis of two triradicals and a tetraradical.

A series of polyradical model systems **60**, **61** and **62** with well-defined inter-spin distances ranging between 2.2 and 3.8 nm, mimicking geometries and aggregation states of biological complexes, were synthesised [27]. Triradicals with different symmetries together with a planar tetraradical were obtained in a convergent reaction scheme based on Sonogashira cross-couplings between aryl halide spacer building blocks and the spin-label **2**, as reported in Scheme 9.

The synthesis was based on the Sonogashira coupling of the spin-labelled spacer **63** with aryl halides **64**, 1,3,5-triiodobenzene or 1,2,4,5-tetraiodobenzene. The bent biradical **40** (constituent biradical of **61**) was the product of the cross-coupling between 1,3-diethynylbenzene and **65**. **39**, instead, was isolated as a side product of the competing Glaser reaction of **63** during its Sonogashira couplings with aryl halides. The systems have been used to scrutinise a PELDOR-based method for counting the number of interacting monomers and test the accuracy and limitations. Additionally, their restricted flexibility allowed quantitative distance measurements including the first resolution of three distances within a single molecule where two distances differed by less than 0.5 nm in **62** [27].

**Figure 8:**
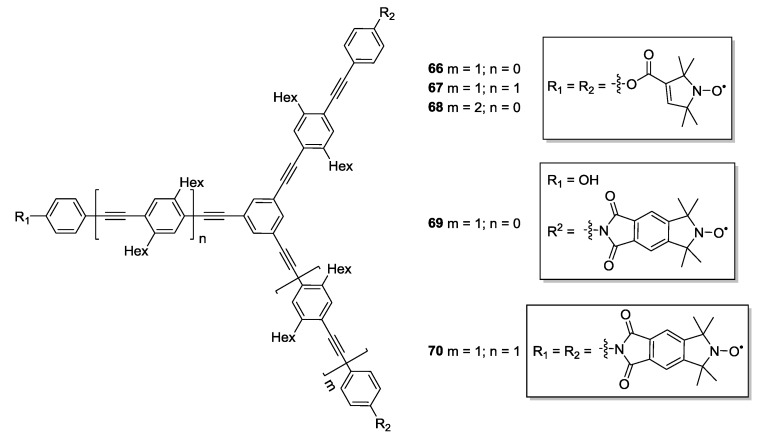
Multi-spin systems with different symmetries.

Multi-spin model systems **66**–**70** (Figure 8) displaying different symmetries and geometries were reported by Jeschke *et al*. [26]. These molecules were used for the isolation of the spin-pair contribution from the multi-spin PELDOR signal. The synthetic strategy is similar to the one previously reported earlier by Godt *et al*. [38]. **3** or **4** are used as spin-labels, as reported in Figure 8. 

Compounds **21** and **67** have been measured at both X- and W-band to quantify the orientation selection effects on distance measurements in both bi- and tri-radicals [26,79]. The mutual orientations and their dynamics could be predicted from simple geometric models and verified by orientation selective PELDOR experiments. Measurements on **38** and **69** allowed calibration of orientation selection experiments at Q-band (34 GHz) frequencies [80].

**Scheme 10:**
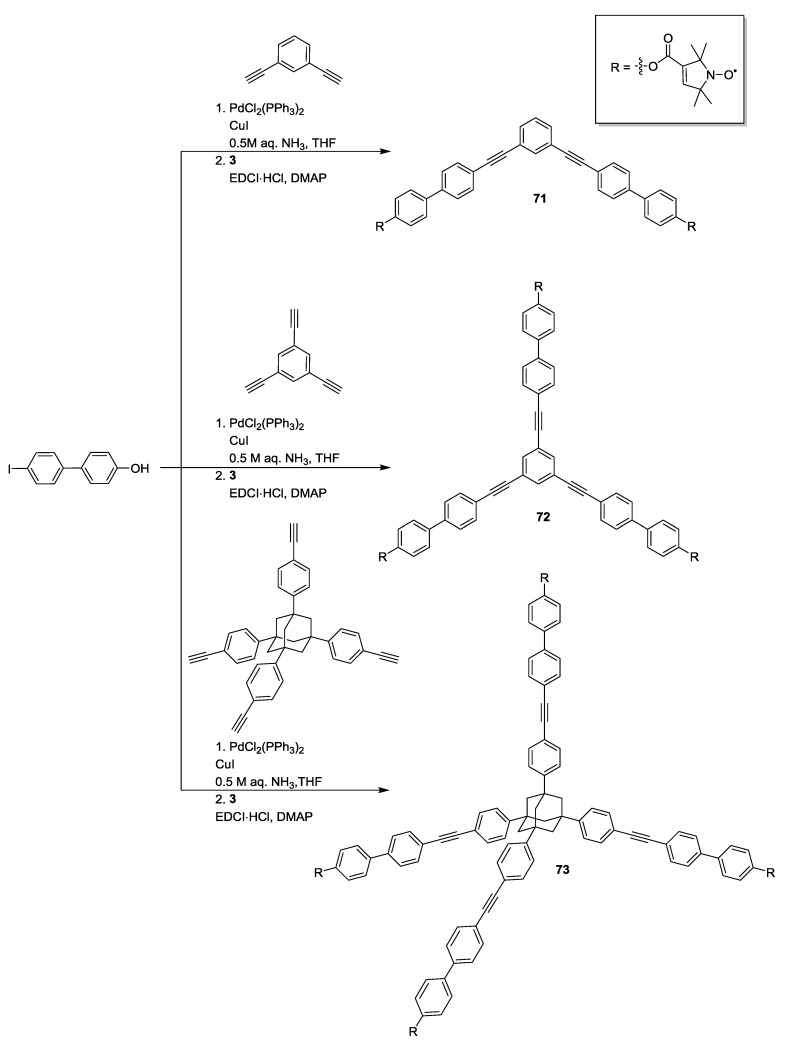
Modular approach for synthesis of polyradicals.

Recently a short synthetic route for the synthesis of symmetric bi-, tri- and tetra-radicals **71**–**73** has been introduced [37]. The modular approach, as reported in Scheme 10, involved the Sonogashira cross-coupling of 4-hydroxy-4'-iodobiphenyl to core building blocks with two to four terminal alkynes using aqueous ammonia as a base. The cross-coupling gave the bis-, tris- and tetrakis-phenols. Esterification reactions using spin-label **3** were performed with EDCI·HCl and DMAP as activating agents. This new approach does not require the use of protecting groups and allows straightforward purification.

### 3.3. Metal Ions

A significant number of biological systems bear catalytically active or structurally relevant metal centres. These metal centres are frequently paramagnetic in one or more functional states of the system or can often be substituted by a paramagnetic one [81,82]. Native metal ion binding sites in biologically relevant systems are an interesting target for distance measurements. PELDOR in particular has been proven to be an accurate tool for measurement of distances not only between nitroxides [19], but also between paramagnetic metal centres [5,52,83] and paramagnetic cofactors [84]. Systems containing a single paramagnetic metal centre require the presence of at least one further spin centre for inter-spin distance measurements. The second paramagnetic centre can be introduced by SDSL using nitroxide radicals [52].

**Scheme 11:**
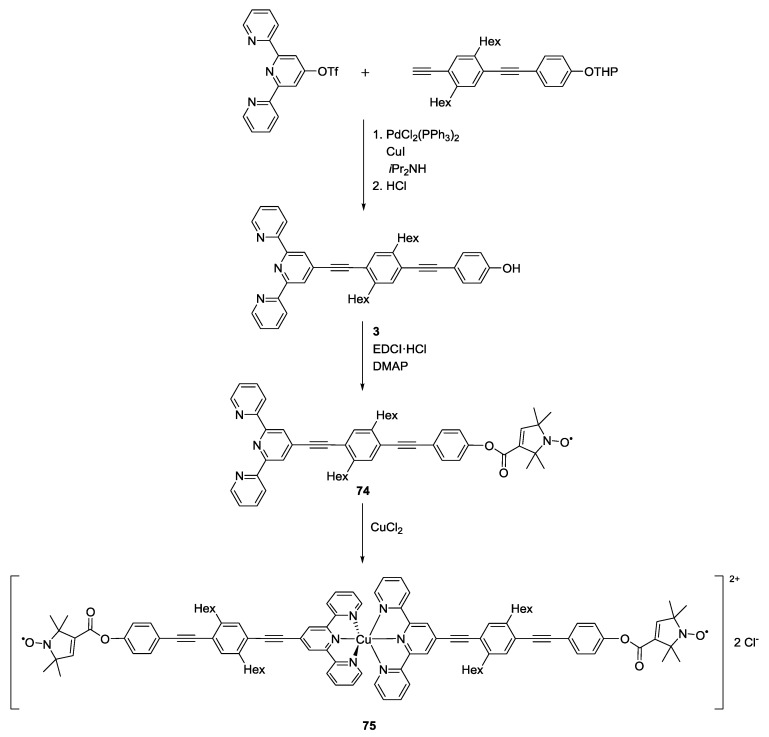
Synthesis of a rigid Cu(II) bisnitroxide system.

Model systems containing two metal centres or a metal centre and a nitroxide radical have been synthesised to test the potential of pulsed EPR distance measurements in these systems. The design commonly involves the synthesis of a chelating ligand, often bearing a rod-like spacer to a second ligand or a stable radical, followed by coordination to one or more metal centres. In contrast to nitroxide spin-labelling, the paramagnet is most commonly introduced into the ligand in the very last step. This can often be performed by dissolving the salt containing the metal ion in water and suspending the ligand in this solution. Distance measurements involving copper(II)-nitroxide [51], copper(II)-copper(II) [82], gadolinium(III)-gadolinium(III) [49], cobalt(II)-nitroxide [73] and iron-sulfur clusters and nickel-iron centre pairs [5] have been reported. Most paramagnetic metal ions possess challenging EPR properties such as huge line widths, fast relaxation times [85], pronounced anisotropies and strong ESEEM effects. As for the previously reported nitroxide-nitroxide yard-sticks, model systems have paved the way for understanding and optimising the use of transition metals for pulsed EPR distance measurements. Narr *et al*. [51] proved that contribution of the Cu(II)-nitroxide coupling could be separated from the nitroxide-nitroxide coupling in the three-spin system given in Scheme 11.

A rigid terpyridine spacer-ligand was esterified to **3** to yield the spin-labelled terpyridine-ligand **74**. Two equivalents of this ligand were coordinated to Cu(II) ions. This design yielded **75** which exhibits a well-defined coordination geometry around the Cu(II) centre. The combination of density functional theory (DFT) calculations and force-field structures allowed modelling of the distance between the paramagnetic centres. The nitroxide-nitroxide distance was observed by choosing frequencies coinciding with the nitroxide spectrum while the Cu(II)-nitroxide distance could be measured by changing one frequency to make it excite the spins centred on Cu(II) exclusively. This was an early experiment to address specific distances between different spin centres by spectral selection.

The model system **76**, containing both a metal ion and a single nitroxide, was synthesised to evaluate the achievable accuracy of distance measurements between these two paramagnetic centres [50]. Effects of orientation selection, conformational flexibility and spin density distribution were quantified. The porphyrin moiety resembles binding motives found in biological systems and introduces moderate delocalisation of spin density into the ligand.

**Scheme 12:**
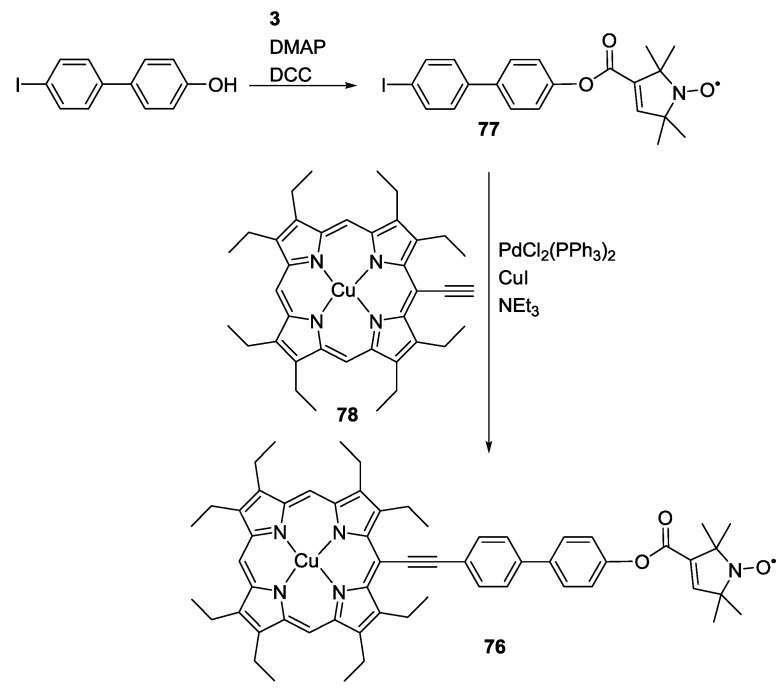
Synthesis of Cu-nitroxide “yardstick”.

The synthesis of the model system (Scheme 12) involved firstly Steglich esterification of 4-hydroxy-4′-iodobiphenyl to **3** to form **77** and subsequent Sonogashira cross-coupling to an alkyne functionalized Cu(II)-octaethylporphyrin **78**. The alkyne functionality was introduced by a sequence of formylation of Ni(II)-octethylporphyrin, Wittig reaction to form the *meso*-chlorovinyl-porphyrin, metal exchange via the free base porphyrin and elimination of hydrochloric acid. Separation of the electron-electron exchange coupling from the dipolar coupling could be achieved by the combination of simulation and experiment. It was shown that Cu(II)-nitroxide distance measurements are feasible in the presence of small through-bond exchange couplings. 

**Scheme 13:**
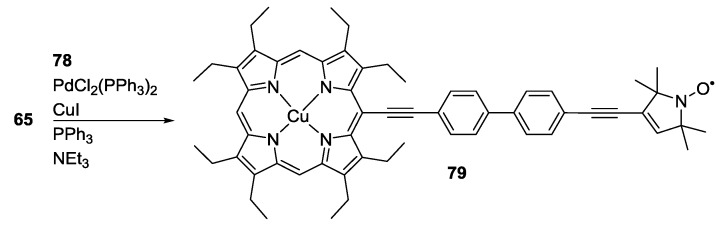
Synthesis of Cu and Ni-based models with **2** used as spin-label to introduce *J* coupling.

The similar system **79** spin-labelled with **2** instead of **3** was scrutinised shortly afterwards [86]. The introduction of the second alkyne bond creates a conjugated bridge which would be expected to induce an increase in *J*. The synthetic path based on coupling between **65** and **78**, reported in Scheme 13, is similar to the one reported for **76**.

The dipolar oscillations were much stronger damped than those for the experiment on **76** in which the conjugation is disrupted by the ester linkage. This damping could be quantitatively attributed to a significant electron-electron exchange coupling by simulations. The extracted distance was validated against crystallographic data obtained on the structurally homologous Ni(II) compound. 

**Scheme 14:**
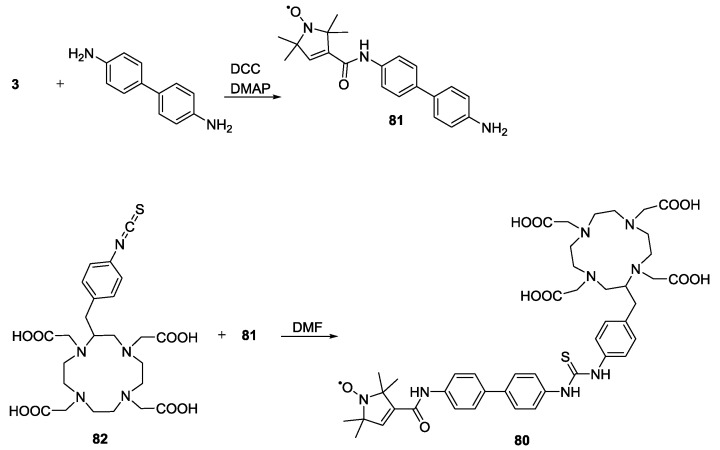
Synthesis of ligand for dipolar relaxation enhancement.

Pulsed EPR distance measurements can only access a specific range of distances due to technical limitations. Long distances require a long observation time window to fully resolve the dipolar oscillation for which slow spin-relaxation would be required. For short distances the pulse excitation bandwidth is too narrow to excite the dipolar coupling. Jäger *et al*. [87] proposed that the long distance limit could be extended by quantifying relaxation enhancement of a slowly relaxing spin system, such as a nitroxide radical, coupled to a fast relaxing electron spin, like a paramagnetic lanthanide ion. To validate this approach to distance measurements the model ligand **80** reported in Scheme 14 was synthesised.

Ligand **80** was used to produce the La(III) and Dy(III) complexes. The synthesis of the ligand involved forming the amide of **3** with benzidine. The monoamide product **81** was then connected to isothiocyanate **82** forming a thiourea-linkage. The measurements allowed estimation of the spin-spin distance from the relaxation enhancement [87].

In principle, distance measurements at high field provide higher sensitivity. This can be exploited by reducing measuring times when compared to X-band or by reducing the amount of sample needed. Nitroxide radicals are not the ideal spin-labels for high field measurements as their spectral width increases with magnetic field. Thus, the proportion of spins excited by the microwave pulses and therefore the signal commonly reduces with magnetic field. Gd(III) ions, on the other hand, have spectral widths that decrease with magnetic field. Thus, they provide a feasible alternative for performing distance measurements at high field. New spin-labels bearing paramagnetic Gd(III) ions have been recently designed and successfully used for labelling of biological systems [35].

**Scheme 15:**
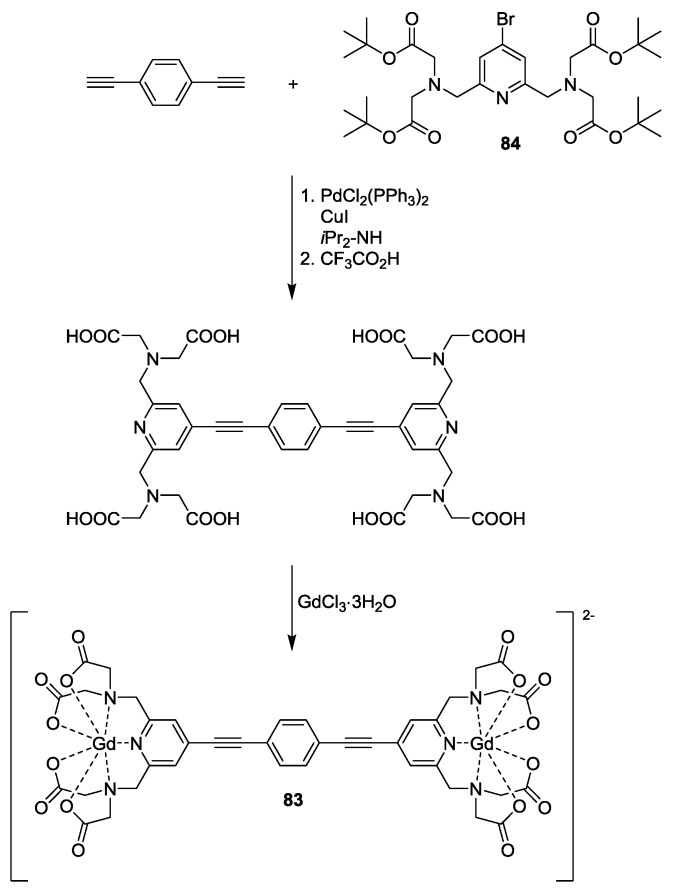
Synthesis of rigid bis-Gd(III) model complex.

To study the potential of Gd(III)-based spin-labelling model system **83** with a rigid bridge was synthesised via Sonogashira cross-coupling of a 1,4-diethynylbenzene to two equivalents of pyridine-based chelator **84** [49]. Hydrolysis followed by coordination to Gd(III) gave the wanted bis-gadolinium system **83** (Scheme 15). This work demonstrated the potential for gadolinium as an alternative spin-label to nitroxide radicals to perform distance measurements at high field, as they provide high sensitivity and present minor orientation selection effects.

**Figure 9:**
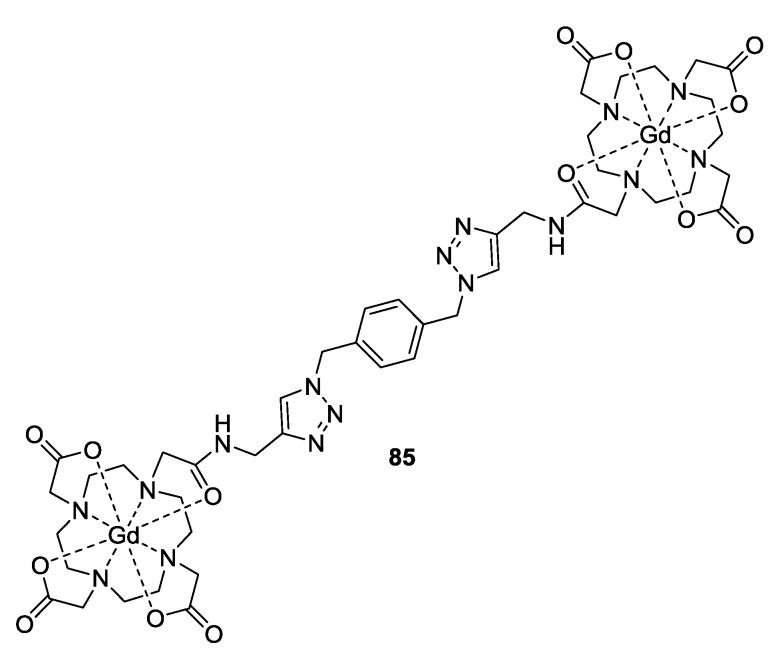
Structure of flexible Gd(III)-based model complex.

Another example for Gd(III)-Gd(III) distance measurements has been reported by Potapov *et al*. [48]. The synthesis of model system **85** (Figure 9) with a flexible bridge was seen as an appropriate reference for biological systems. Flexible systems are considered to be challenging for distance measurements as their distance can be broadly distributed and the corresponding dipolar oscillations will be fully damped in the primary data. The magnetic properties of Gd(III) should in principle allow measurement of longer PELDOR distances between Gd(III) ions than between nitroxides.

Fast relaxing Gd(III) was shown to be complementary to established nitroxide radicals for distance determination in rigid and flexible systems [48]. Gd(III)-nitroxide distance measurements were first performed employing **74** ligated to the metal ion [88]. 

## 4. Applications

### 4.1. Polymer Degradation Studies *via* Distance Measurements

**Figure 10:**
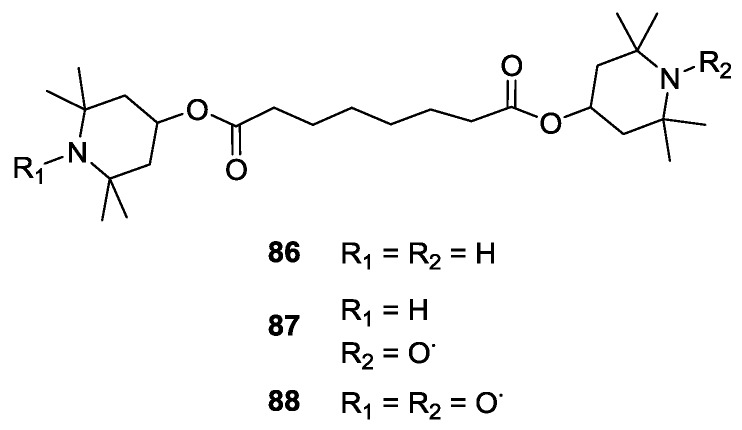
Structure of the Tinuvin 770 (**86**) and its oxidation products.

EPR techniques have been extensively used for study of radical-driven polymer degradation [89]. The degradation process can be prevented via addition of hindered amine stabilisers (HAS), such as Tinuvin 770 (**86**). HAS can form stable nitroxide radicals such as mono- and bi-radical **87** and **88**, respectively. These nitroxides can scavenge reactive radical intermediates in radiation- or light-induced polymer degradation. Jeschke *et al*. [89] used distance measurements and EPR imaging on stabiliser-doped poly(acrylo-nitrile-butadiene-styrene) exposed to heat treatment for studying the spatial distribution of nitroxide radicals formed from stabiliser Tinuvin 770 **86**, as reported in Figure 10.

Model free analysis of distance measurements overestimated the radical concentration. However, combining the analysis with the imaging data a consistent analysis was achieved. The amount of biradical formed was found to be negligible [89].

### 4.2. Biradicals as DNP NMR Polarising Agents

Biradical polarising agents have become an important aspect in dynamic nuclear polarisation (DNP) NMR at liquid nitrogen temperatures [90]. DNP improves the signal-to-noise of NMR experiments by creating an enhanced, non-Boltzmann polarisation. Biradicals have been found to yield large enhancements in cryogenic magic angle spinning (MAS) DNP. This is attributed to the significant dipolar coupling in biradicals as compared to the smaller average dipolar interaction arising from statistical distributions of monoradicals. A water/glycerol solvent system is widely used for DNP analyses of proteins as it combines solubility, cryoprotective and spectroscopic properties. Design of biradicals requires them to have a large dipolar coupling and to be soluble [91] and stable. Rigidity plays a particularly important role as the signal enhancement has been proven to also depend on the relative orientation of the radicals [92]. 

**Scheme 16:**
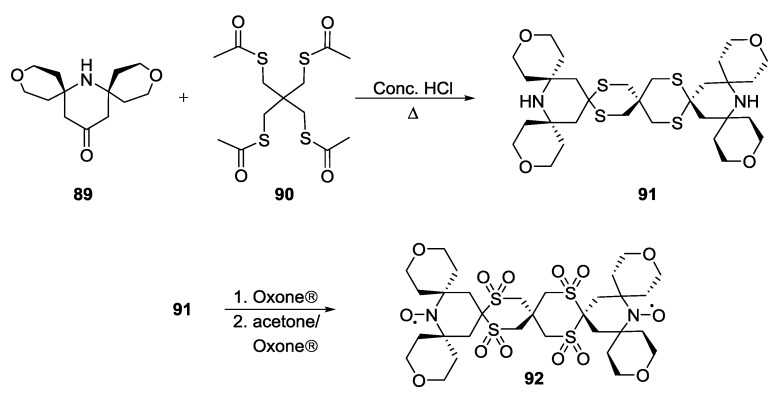
Synthesis of water-soluble biradicals for DNP.

Highly soluble biradicals were synthesised by Kiesewetter *et al.* [93]. The synthesis (Scheme 16) was based on the formation of S,S-ketals from substituted piperidones **89** and **90** to give the intermediate **91**. Treatment of **91** with Oxone® gave access to rigid biradical **92**.

Further biradicals with even larger substituents [94] proved to increase electron spin relaxation times and solubilities which are both crucial for DNP performance.

## 5. Conclusions

The synthesis and application of biradical and polyradical model systems bearing different spin-labels, such as nitroxide and trityl radicals together with metal centres, such as Cu(II) and Gd(III), have been reviewed here.

The desired magnetic, chemical and physical properties of the model systems to be synthesised play an important role in the design of these molecules. The need for specific properties, such as flexibility, solubility, favourable relaxation times, nanometre inter-spin distances and synthetic accessibility, led to the development of a variety of methods for the synthesis of these compounds.

They have been used for calibration of new EPR measurements, such as dead-time-free PELDOR, DQC or SIFTER, together with the study of system-specific properties, such as flexibility, multi-spin and orientation selection effects. Model systems play a key role for the study of complex unknown systems as their experimental behaviour can be reasonably well predicted. These measurements yield valuable insights which can be transferred to cutting edge research on biological systems or materials.
